# Hearing loss symptoms and leisure noise exposure in university students in Barranquilla, Colombia

**DOI:** 10.1590/2317-1782/20212020379

**Published:** 2021-11-15

**Authors:** Dellanira Isabel Escobar-Castro, Michelle De Jesús Vivas-Cortés, Cindy Paola Espinosa-Cepeda, Alberto Mario Zamora-Romero, Martha Elena Peñuela-Epalza

**Affiliations:** 1 Programa de Medicina, Universidad del Norte - Barranquilla (Atlántico), Colombia.; 2 Departamento de Salud Pública, División Ciencias de la Salud, Universidad del Norte - Barranquilla (Atlántico), Colombia.

**Keywords:** Noise-Induced Hearing Loss, Exposure to Noise Pollution, Noise Measurement, Threshold Limit Values, Hypoacusis, Recreational Personal Listening Devices, Ringing-Buzzing-Tinnitus, Tinnitus, Young Adults

## Abstract

**Purpose:**

The aim of this study was to investigate the total weekly exposure to leisure noise among university students and to assess its association with self-reported symptoms of hypoacusis.

**Methods:**

This is a cross-sectional survey. An online questionnaire based on the “Noise Exposure Questionnaire”, plus 11 questions regarding hearing loss were sent to 730 randomly selected students. Participants self-reported time spent on different leisure noise activities and their subjective evaluation of the loudness of these activities, converted into equivalent noise levels, were used to estimate weekly noise exposure levels that were compared to occupational noise limits (> 85 dBA = hazardous). Inference statistics was applied to relate hearing symptoms and “likely or having some degree of hearing loss” with hazardous weekly leisure noise exposure levels.

**Results:**

Ninety-three percent of the participants reported at least one hypoacusis symptom. The most frequent sound-related ear symptom was tinnitus (72%). Fifty-five percent of the individuals presented weekly exposure to noise >85 dBA. Symptoms of hearing loss were more prevalent in those exposed to weekly noise levels >85 dBA.

**Conclusion:**

This study suggests that there may be hearing loss caused by exposure to high levels of leisure noise in a large part of the study population. Health promotion of hearing conservation should be emphasized at university level. Objective repeated measurement of hearing acuity should be part of integral health services for the youth population.

## INTRODUCTION

Hearing disorders are part of the group of chronic diseases due to their slow evolution and long duration. There is evidence of the negative impact of hearing loss on people's quality of life, causing social isolation, depression, changes in cognitive performance, and low self-esteem ^(1-2)^.

Hearing disorders constitute an important public health problem because of their increasing prevalence in the world order and the economic impact they generate on health care systems and the economy in general ^(3-4)^. According to recent estimates of the World Health Organization (WHO), approximately 466 million people worldwide suffer from disabling hearing loss. By 2050, one in 10 people will suffer from disabling hearing loss. Likewise, 1,100 million young people aged 12-35 years are at risk of suffering hearing loss as a result of exposure to noise in recreational contexts ^(5)^.

In Colombia, according to estimates of 2016, 17% of the population had hearing problems, with a marked upward trend in the age groups of 19-26 and 6-9 years. Among individuals aged 25-50 years, the prevalence of hearing loss was 14%, and only three out of 10 people have sought specialized assistance ^(2)^.

Consumption of tobacco, alcohol or psychoactive substances, environmental pollution, high level of noise in urbanized areas, and exposure to environmental or occupational toxins and recreational noise are preventable causes of hearing damage in the population. In young people, exposure to recreational noise predominates because of prolonged and frequent use of sound reproducing speakers, personal audio and multimedia content devices, and headphones, as well as a result of attendance to places with excessive noise, such as discos and concerts ^(6-7)^. A study highlighted the use of personal audio devices, for longer periods and at a very loud volume, among the Hispanic population of adults and adolescents ^(8)^.

The association between recreational noise and hearing loss in young people has been studied in various regions of the world ^(9-13)^, and its consequences on hearing impairment in later life are already evident in the Eurotrak 2009-2015 study ^(14)^ and, more recently, in the EuroTrack 2020 report, carried out jointly by the European Federation of Hard of Hearing People (EFHOH), European Association of Hearing Aid (AEA), and European Hearing Instrument Manufacturers Association (EHIMA). According to estimates from the latest report, the average self-reported hearing loss is 11.1% for Europe as a whole, where 20% of the population is ≥64 years old. Furthermore, it is noted that 40% of the people who stated that they use headphones as a method for reproducing audiovisual content present hearing impairment ^(15)^.

It is known that the degree and extent of hearing damage are closely related to the sound pressure level, its frequency and duration, as well as with individual genetic susceptibility ^(16)^. Noise-induced sensorineural hearing loss is usually accompanied by symptoms such as tinnitus, decreased discrimination capacity, and sound distortion ^(17)^, which are manifestations that can be evaluated by subjective methods.

Despite the relevance of the previously discussed topics, exposure to leisure noise has not been sufficiently studied in the young adult university populations in Latin American cities. Colombia has had defined policy guidelines for the promotion and comprehensive management of hearing and communicative health since 2014, with programs that emphasize actions for health promotion, primary prevention, early detection, and treatment ^(18)^. Under these guidelines, this study investigated the participation of undergraduate students in noise-producing leisure activities in order to analyze the relationship between levels of weekly exposure to recreational noise and self-reported hearing loss symptoms in university students in the city of Barranquilla, Colombia.

## METHODS

This study was approved by the Health Research Ethics Committee of “Universidad del Norte” according to act no. 177 of August 30, 2018. All participants signed an Informed Consent Form (ICF) prior to study commencement.

A cross-sectional, observational study was carried out to assess the recreational factors of exposure to noise and their association with the report of hearing loss symptoms in university students aged 18 to 29 years enrolled in undergraduate programs at a university in the city of Barranquilla during the first semester of 2019. The covariates studied were sex and living in a noisy residential area.

The sample was calculated from a total eligible population of 13,431 students, with an estimated proportion of hearing loss in young people of 17% ^(2)^, a precision of ±3, a confidence level of 95%, and a design effect of 1.2. To the estimated number of 692, 5% was added to provide for eventual need to discard questionnaires because of the previous existence of a basic hearing problem, and thus the total sample was composed of 730 students. Individuals were randomly selected, stratified by academic department and the courses they were enrolled. An invitation message with the link to the online questionnaire was sent to students and academic program coordinators in order to stimulate participation.

The questionnaire, developed in Google Forms, was structured in several sections. The first showed the terms of the ICF followed by a question and an approval box, which if not verified, prevented the student from continuing to the questionnaire. There was also a question about previously diagnosed hearing impairments (study exclusion criterion) and some related to general data (age, sex, academic program).

The second section included the “Noise Exposure Questionnaire” (NEQ) - a translated version adapted to Spanish from its original version in English ^(19)^, validated by expert judgment ^(20)^. The NEQ is composed of two parts: Part A, which recorded the participation in leisure activities, the daily time and the number of days per week dedicated to each of them, and the subjective perception of noise on a scale from 1 “very quiet” to 5 ”very noisy “; Part B, which inquired about symptoms (tinnitus, earache, and temporary hearing loss caused by noise).

The third section of the questionnaire includes the 11 items of the auditory questionnaire developed by the company Phonak^®^ (available at https://www.yumpu.com/es/document/read/25302942/cuestionario-auditivo-phonak) for the self-assessment of the possible presence and degree of severity of hearing loss.

### Measurement of noise exposure

Following the method proposed by Jokitulppo et al ^(19)^ for the transformation of the subjective assessment of respondents on the noise level of the activities evaluated in the questionnaire, the equivalent continuous sound level (LAeq dB) established in the international literature was considered (Table 1). The value in equivalent decibels of maximum continuous noise in each activity was assigned the maximum score of “5” (very noisy) recorded in the questionnaire. Then, based on this value, we proceeded to reduce 10 decibels for each point below 5. For example, in the category of personal audio devices, the maximum exposure value (5, very noisy) is equivalent to 100 decibels; in other words, for the lower values ​​answered in the questionnaire, the noise equivalence in decibels would be the following: 4 = 90 dB, 3 = 80 dB, 2 = 70 dB, and 1 = 60 dB. Subsequently, the average weekly exposure to recreational noise was calculated and compared with the permissible limit for occupational noise exposure, established at 85 dBA, adopted in Colombia ^(21)^.

**Table 1:** The equivalent sound levels of leisure time activities.

**Activity**	**Variation range**	**LAeq Max**	**Reference**
Playing in band	90-135	105	MCR, 1986; Salamivalli, 1990; Drake-Lee, 1992
Listening clasical music	75-114	105	Axelsson et al, 1981a; Jansson y Karsson, 1983; Royster y Royster, 1991;
			Royster y Royster, 1991;
Video arcades	73-111	100	
Home stereos (loud speakers)	70-100	100	Axelsson et al, 1981a; Salmivalli, 1990
Home stereos (headphones)	85-120	100	Axelsson et al, 1981a; Salmivalli, 1990
Personal stereos	53-115	100	Kurss y Findlay, 1974; Catalano & Levin, 1985;
			Lees et al, 1985; Wong et al, 1990; Clark, 1991; Airo et al, 1995
Motor sport	70-112	105	Axelsson et al, 1981a; Clark, 1991
Disco and pop-concerts	84-125	105	Fearn, 1972; Ulrich et al, 1974; Axelsson et al,
			1981a; MRC, 1986; Salmivalli, 1990; Göthe, 1992
Home tools	60-115	105	Axelsson et al, 1981; Salmivalli, 1990; Clark, 1991
Shooting		105	Estimado

Source: Jokitulppo et al.^(19)^

**Caption:** LAeq = The equivalent sound level

### Measurement of the degree of hearing loss

To classify the possible degree of hearing loss, based on the 11 questions of the hearing questionnaire, for each affirmative answer, 1 point was assigned (range from 0 to 11 points). After counting the total number of affirmative responses to questions per participant, two groups were formed: group “with probable hearing loss” of some degree (4 or more points) and group “without suspicion of hearing loss” (0-3 points).

The association between the symptoms of hearing loss from the NEQ questionnaire and the possible degree of hearing loss with the equivalent recreational noise exposure level (“>85 dB” *vs*. “<85 dB”) was evaluated using the odds ratio, the 95% confidence interval, and the statistical significance established at *p*<0.05, using the Chi-squared test (*X^2^* ). The analysis was carried out in the Epi-info 7.2 software.

## RESULTS

All students invited to participate answered the questionnaire. Of the 730 surveys, 18 were excluded due to report of previous hearing problems, thus 712 individuals were included in the analysis. Just over half of the respondents were female (59.6%) and there was predominance of the 18 to 27-year age group (87%) for both sexes, with no significant differences in the distribution by age and sex (*p*>0.05). 25.2% were from Engineering programs, 18.3% from Health Sciences, 18.1% from Humanities, 14.8% from Business school, 12.29% from Law and Political Sciences, 5.3% from Architecture, 3.7% from Basic Sciences, 1.2% from Education, and 0.7% from Music. 50.28% (358/712) lived in a noisy residential area.

### Exposure to recreational noise

**Figure 1:**
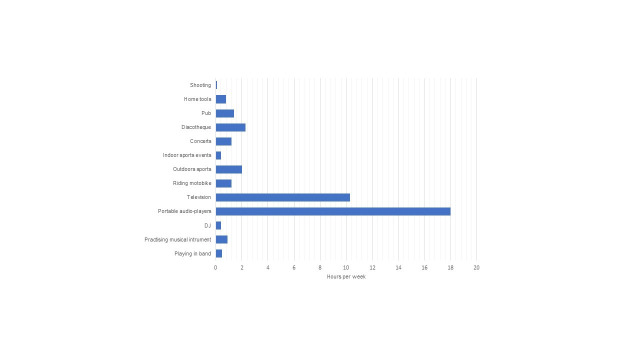
The participants’ time consumed (average hours per week) of different leisure activities.

**Figure 2:**
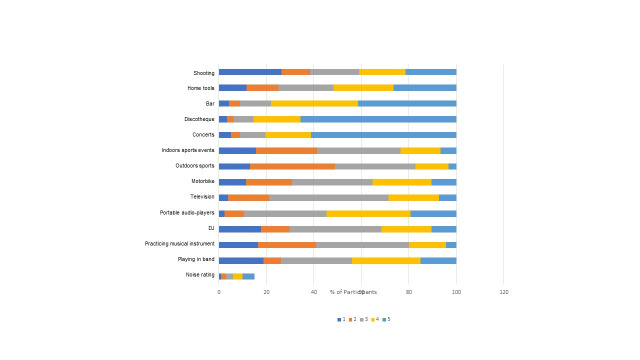
Distribution of the participants loudness ratings of leisure time activities on a scale from 1 “quiet” to 5 “very loud”.

The recreational activities evaluated in which the respondents spent the most time per week were use of portable devices (18 h), TV watching (10.3 h), and going to discos (2.2 h) (Figure 1). The leisure activities perceived as loudest by university students were discos, concerts, bars, shooting, and the use of personal portable audio devices (Figure 2).

**Figure 3:**
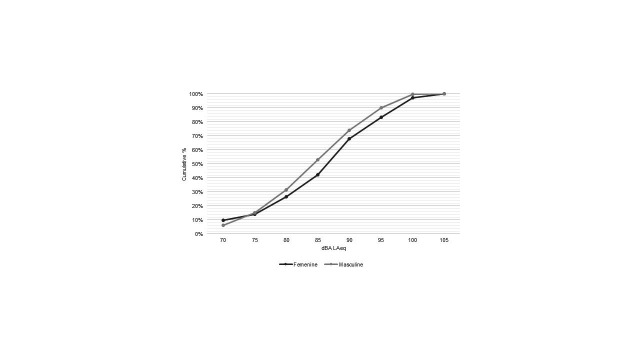
Distribution of weekly noise exposure by sex. The results have been calculated for participants exposure to all types of noises, the duration of each activity and the estimated level for these activities.

The weekly exposure to noise calculated for the total sample, in relation to all the activities, the time devoted to each of them, and the estimated noise intensity for each activity show that the median exposure was 87 dBA. When analyzing the distribution of exposure by sex (Figure 3), it was found that 58% of women, in contrast to 47% of men, are exposed to noise levels above the risk limit of 85 dBA established in the Colombian standard ^(20)^, which reveals significant differences (*p*<0.05).

### Symptoms of hearing loss

Of the symptoms investigated in the NEQ questionnaire, ringing in the ears was the most reported (72%), followed by earache (44%), and temporary hearing loss (23%). Table 2 shows that exposure to noise levels above the risk threshold for hearing damage was significantly associated with the presence of tinnitus and earache (*p*<0.05).

**Table 2:** Association between hearing symptoms and noise exposure levels

	**Ringing in ears**			**Pain**			**Temporary hearing loss**		
	**n (%)**	**OR (IC_95%_)**	**Valor p** *	**n (%)**	**OR (IC_95%_)**	Valor p*	**n (%)**	**OR (IC_95%_)**	Valor p*
***>85 dB***	309 (60)	1.78 (1.28-2.4)	<0.05	200(63)	1.73 (1.27-2.3)	<0.05	93(56)	0.99 (0.7-1.4)	>0.05
**≤85 dB**	206 (40)			115(37)			73(44)		
**Total**	515 (100)			315(100)			166(100)		

* Statistic: X^2^

**Caption:** n= number of cases; OR= *odds ratio*; IC= confidence interval

Table 3 shows that of the 11 hearing loss symptoms evaluated through the Auditory Test, the five most frequently reported were “problems understanding conversations when there is noise in the environment or people speaking” (55%), “need to ask others to repeat” (49%), “not understanding what is being said when driving or in a noisy environment” (46%), “feeling stressed or tired when listening to something for a long time” (40%), and “setting the TV volume higher to hear well compared with others” (39%).

**Table 3:** Frequency of hypoacusis symptoms among total participants (Hearing Test) and number of symptoms per participant

.	n (%)	IC 95%
**Hypoacusis Symptoms:** Difficulties for understanding in noisy or crowded environments	394 (55)	51.6-59.0
Need to ask others to repeat what they have said	353 (49)	45.8-53.3
Difficulties for understanding when in a car or in a noisy environment	327 (46)	42.2-49,6
Feel stressed or tired after hearing something for a long period of time	285 (40)	36.3-43.7
Turn up the TV volume louder than others to hear.	276 (39)	35.142.4
Think others are mumbling or talking not clearly	245 (34)	30.8-37.9
Needs to be very close to others to hear what they say	209 (29)	25.9-32.7
Difficulty hearing others who are not in front	166 (23)	20.1-26.5
Avoid attending parties or events due to hearing problems	148 (21)	17.7-23.8
Asked about hearing problems	133 (19)	15.7-21.6
Difficulties to identify the source of the sound.	105 (15)	12.0-17.4
**Number of hypoacusis symptoms per participant:**		
1 a 3 symptoms	306 (43)	39.3-46.7
4 a 6 symptoms	263 (37)	33.3-40.5
7 a 11 symptoms	93 (13)	10.5-15.6
0 symptom	50 (7)	5.5-8.9

**Caption:** n= number of cases; IC= confidence interval

The classification of the estimated degree of probable hearing loss, according to the number of symptoms (Table 3), shows that just under half are located in the group of symptoms 1-3, considered as “without suspicion of hearing loss” (43%), while the rest classify for the group “with probable hearing loss of some degree”.

### Demographic variables and probable hearing loss

Although women were 1.3 times more likely to present probable hearing loss than men, no significant association was found (*p*>0.05). Likewise, “living in a noisy residential area” was not associated with the possibility of having probable hearing loss (*p*>0.05) (Table 4).

**Table 4:** Association between likely hearing-loss and sex, zone of residence and level of recreational noise exposure

	Likely hearing loss	No hearing loss	**OR (IC_95%_)**	**Valor p** *
	n= 352	n=360		
Sex				
				
Feminine	222	203	1.32 (0.97-1.78)	0.069
				
Masculine	130	157		
				
Noisy neighborhood				
Si				
No	176	182	0.9 (0.72-1.31)	0.882
				
	176	178		
				
Recreational noise exposure level				
> 85 dBA				
	217	182	1.57 (1.16-2.11)	0.002
≤ 85 dBA				
	135	178		

* Statistic: X^2^

**Caption:** OR= *odds ratio*, IC= confidence interval

### Exposure to recreational noise and estimated degree of probable hearing loss

Students who were exposed to weekly levels of recreational noise above the permissible limit had a 1.57-time (95% CI = 1.16-2.11) greater chance of having four or more hearing loss symptoms; that is, probable hearing loss of some degree (Table 4). This association was maintained in the analysis adjusted for sex (OR = 1.53; 95% CI = 1.12-2.09).

## DISCUSSION

To the best of our knowledge, this is the first study addressing the prevalence of self-reported hearing loss symptoms in relation to exposure to recreational and environmental noise conducted with undergraduate college students aged 18-29 in a capital city of the Colombian Caribbean region.

A relevant finding of the study is the time spent weekly in noise-producing recreational activities, in particular, the use of personal audio devices and television, which is similar to that reported for young people in Europe ^(12,18-19)^, Asia ^(9)^, the United States ^(7)^, and Latin America ^(8,22)^.

It has been reported that personal audio devices generate sound pressure levels as high as 124 dBA ^(12-13,23)^. However, only half of the young university students investigated perceive this activity as very noisy or not very noisy, in contrast to what was reported by young Asians ^(9)^.

Likewise, the attendance to nightclubs for 2.3 h a week, declared by the participants, constitutes a very important noise exposure factor in the environment, not only because it generates higher intensity levels ^(21)^, but also because of the possibility of having been underestimated in the study, once the NEQ questionnaire inquires about weekly attendance, but not for longer periods.

Unlike young Europeans ^(18-19)^, exposure related to playing in a music group was infrequent in the university population surveyed, probably because music students were underrepresented in this study, as this was a recently opened program at the university where the study was conducted.

The median total weekly exposure to noise in this study (87 dBA) was 14 dBA higher than that registered for the Nordic adult population ^(24)^ and 2 dBA higher than that estimated for Spanish adolescents ^(19)^. Just over half of the population studied is exposed to a level of recreational noise above the internationally established risk limit, which implies a risk of suffering from hearing loss in the short, medium or long term. This frequency is lower than that found in studies conducted in Germany (25%) using a subjective measurement and audiometry as the objective method ^(12)^. However, it coincides with those reported in previous studies conducted with adolescents aged up to 19 years ^(18)^, young Finnish adults ^(24)^, and Spanish adolescents ^(19)^ that used the same methodology. Likewise, it is consistent with the WHO global estimates for adolescents and young people aged 12-35 years ^(25)^. The high percentage of exposure to dangerous levels of recreational noise in different countries suggests that the programs to promote hearing health present deficiencies, despite the existing regulations.

The high proportion of university students with probable hearing loss of some degree found in this study could be explained, in part, by the accumulation of exposure to recreational noise during adolescence. In all cases, the most reported activities (listening to music in audio devices and watching television in high volume) are habits that tend to remain or even increase during life. According to a 4-year follow-up audiological study carried out with adolescents in the city of Córdoba, Argentina, the average hearing threshold level tends to increase over time, in parallel with the increase in participation in musical recreational activities and attendance to discos and concerts ^(6)^.

Tinnitus, identified by the perception of ringing in the ears, was the most reported auditory annoyance in the present study, and thus deserves particular attention, as hearing impairment is its main risk factor ^(26)^. The findings of a systematic review ^(27)^ indicate that there is controversy in the effects of tinnitus on working memory and attention, as well as a high probability of bias in published studies. However, these functions are of paramount importance in the academic performance of university students and, therefore, they should be addressed in future research.

The analysis of factors associated with the presence of isolated hearing loss symptoms and simultaneous presence of several symptoms suggestive of probable hearing loss of some degree indicates that there were no significant differences in the presence of these hearing loss symptoms among the participants who declared to be exposed and not exposed to residential areas near construction sites or heavy traffic. This finding may be explained by the noise pollution described in the city of Barranquilla, caused by various sources of noise ^(28)^.

This study provides new evidence of the risk posed by exposure to recreational noise levels above the 85 dBA limit for the young university population by observing that this population has a greater chance of simultaneously presenting several symptoms suggestive of probable hearing loss of some degree. These findings urge the need for the academy to implement promotion and prevention actions. At the university level, these actions are framed in recommendations of international and national policies for health care and promotion. The former action, based on the concept of a healthy or health-promoting university, defined as *“one that incorporates health promotion into its educational and work project in order to foster human development and improve the quality of life of those who study or work there and, at the same time, train them to act as models or promoters of healthy behaviors at the level of their families, in their future work environments, and society in general”* ^(29)^, and the latter action, based on the national guidelines for the promotion and comprehensive management of hearing and communicative health ^(18)^.

A limitation to this study is the ignorance of the true hearing capacity of the participants due to the subjective measurement used. According to reviews on the reliability of self-reported hearing loss surveys, subjective measurements present inaccuracies: they underestimate the presence of hearing loss in older adults and tend to overestimate it in younger adults ^(4)^; therefore, they are insufficient to accurately identify hearing-impaired individuals. However, the questions presented in the auditory questionnaire allow, from the academic setting, knowledge about the different symptoms that could be related to such changes. In particular, questions about difficulty in understanding conversation in noisy environments assist in detecting possible hidden hearing loss or cochlear synaptopathy, which may go unnoticed on an audiogram ^(30)^. Another limitation to this study is the failure to assess the frequency of hearing loss symptoms.

According to the literature review carried out by these authors, there is a lack of publications addressing hearing loss in the university population in Colombia - a situation that limited the possibility of comparing the results obtained in Barranquilla with those of other important cities of the country. In this sense, this study is useful as a precedent for future research where hearing acuity is established by subjective and objective assessment methods and correlated with environmental, recreational and behavioral characteristics. Additionally, it serves as a basis for the development of awareness and education programs in hearing health that promote the adoption of protective behaviors against possible hearing problems in university settings. This study also recommends the screening of hearing through medical examination in students who have just entered university, as well as corrective behaviors to prevent hearing problems.

## CONCLUSION

The subjective estimation of the intensity of exposure to noise and the symptoms of hearing loss in university students serves as screening for the early identification of hearing problems and provides population-based information for decision-making. The high prevalence of auditory symptoms reported in this study reflects the relevance of this problem. Although the university students surveyed identified the loudest recreational and environmental activities, they declared that the use of portable audio devices and going to discos are among their preferred daily activities, which expose them to average intensity levels >85 dB, and thus entails a risk of severe hearing impairment.

This study provides new evidence on the association between prevalence of auditory symptoms and continuous exposure to average noise levels >85 dB in university students. It is necessary to incorporate or intensify the promotion of hearing health in university settings and raise awareness of the behaviors that predispose the development of hearing loss or worsen its basic condition. Simultaneously, it is recommended that more objective early detection programs be carried out through the university welfare services, in coordination with the health services network. In this way, continuity in the care of young people at risk of hearing loss can be facilitated, guaranteeing timely treatment and limiting the damage.
