# Supplementary material for: Hearing loss symptoms and leisure noise exposure in university students in Barranquilla, Colombia
Source: Codas. 2021 Nov 15;34(1):e20200379. doi: 10.1590/2317-1782/20212020379 (PMC9886120; doi:10.1590/2317-1782/20212020379)
Supplement: Translation

# Translation: Síntomas de hipoacusia y exposición al ruido recreativo en jóvenes universitarios, Barranquilla, Colombia

**Authors:** Dellanira Isabel Escobar-Castro, Michelle De Jesús Vivas-Cortés, Cindy Paola Espinosa-Cepeda, Alberto Mario Zamora-Romero, Martha Elena Peñuela-Epalza

**Affiliations:** 1 Programa de Medicina, Universidad del Norte - Barranquilla (Atlántico), Colombia.; 2 Departamento de Salud Pública, División Ciencias de la Salud, Universidad del Norte - Barranquilla (Atlántico), Colombia.

**Keywords:** es, Pérdida Auditiva Provocada por Ruido, Exposición al Ruido, Medición del Ruido, Valores Límites del Umbral, Hipoacusia, Dispositivos Personales de Áudio, Zumbido, Acúfenos, Adultos Jóvenes

## Abstract

**Objetivo:**

Investigar la participación de estudiantes universitarios en actividades de ocio productoras de ruido y la relación entre los niveles de exposición semanal al ruido recreativo y síntomas de hipoacusia auto reportados, en la ciudad de Barranquilla, Colombia.

**Método:**

Se realizó un estudio transversal mediante encuesta virtual, basada en el Cuestionario de Exposición al Ruido, más 11 preguntas de síntomas auditivos, a una muestra aleatoria de 730 sujetos. El nivel de exposición semanal al ruido recreativo se estimó a partir del tiempo dedicado al total de actividades recreativas reportadas por los participantes y su evaluación subjetiva de la intensidad de ruido producida por cada actividad, transformada en niveles de ruido continuo equivalente. La asociación entre la exposición al ruido recreativo y la presencia de síntomas de hipoacusia se analizó mediante la prueba de Chi-cuadrado.

**Resultados:**

El 93% de los participantes manifestó al menos un síntoma de hipoacusia y la mitad reportó cuatro o más. El síntoma más frecuente de hipoacusia asociada al ruido fue el tinnitus (72%). El 55% tenía una exposición semanal de ruido por encima de 85 dBA y en éstos la prevalencia de síntomas de hipoacusia fue mayor (p < 0.05).

**Conclusión:**

Se sugiere la presencia de deterioro auditivo en gran parte la población universitaria, asociado con altas dosis de exposición semanal al ruido recreativo. Es necesario fortalecer los programas de promoción de la salud auditiva en la academia, y desde los servicios de salud, la valoración objetiva y periódica de la audición.

## INTRODUCCIÓN

Las alteraciones auditivas hacen parte del grupo de enfermedades crónicas por su lenta evolución y larga duración. Existe evidencia del impacto negativo de la pérdida de la audición en la calidad de vida de las personas, al causar aislamiento social, depresión, alteraciones en el desempeño cognitivo y baja autoestima^(1-2)^ **.**

Los trastornos de la audición constituyen un problema importante de salud pública debido a su creciente prevalencia en el orden mundial y al impacto económico que generan en los sistemas de atención salud y en la economía en general ^(3-4)^. Según estimaciones recientes de la OMS: alrededor de 466 millones de personas en todo el mundo padecen pérdida de audición discapacitante; en 2050, 1 de cada 10 personas, sufrirá una pérdida de audición discapacitante; asimismo 1100 millones de jóvenes, entre los 12 y 35 años, están en riesgo de padecer pérdida de audición por su exposición al ruido en contextos recreativos^(5)^.

En Colombia, según estimaciones realizadas en 2016, el 17% de la población presentaba problemas de audición, con una marcada tendencia al incremento en los grupos etarios de 19-26 años y de 6-9 años. Entre el grupo de 25-50 años, la prevalencia de pérdida de la audición fue del 14%, y solo 3 de cada 10 personas recurrían a la ayuda de un especialista en el tema^(2)^.

El consumo de tabaco, alcohol o sustancias psicoactivas, la contaminación ambiental, el alto nivel de ruido en las áreas urbanizadas, la exposición a tóxicos ambientales u ocupacionales y el ruido recreativo son causas prevenibles de daño auditivo en la población. En los jóvenes, predomina la exposición al ruido recreativo, debido al uso prolongado y frecuente de parlantes reproductores de sonido, dispositivos personales de audio y contenido multimedia y auriculares; también por la concurrencia a sitios con exceso de ruido, tales como discotecas y conciertos. ^(6-7)^. Un estudio destaca el uso de dispositivos personales de audio, por periodos más largos de tiempo y a un volumen muy alto, entre la población hispana de adultos y adolescentes ^(8)^ .

La asociación entre el ruido recreativo y la pérdida auditiva en jóvenes ha sido estudiada en diversas regiones del mundo ^(9-13)^ y sus consecuencias en el deterioro de la audición, en etapas posteriores de la vida, son ya evidentes en el estudio Eurotrak 2009-2015 ^(14)^ y más recientemente, en el reporte EuroTrack 2020, realizado en conjunción por las asociaciones *Hard of Hearing People* (EFHOH), *Hearing Aid Professionals* (AEA) and *Hearing Instrument Manufacturers* (EHIMA). Según estimaciones del último informe, el promedio de auto-reporte de pérdida auditiva es de 11.1% para toda Europa, donde el 20% de la población es ≥ 64 años; además, se señala que 40% de las personas que manifestaron usar auriculares como método para reproducción del contenido audiovisual presentan deterioro auditivo^(15)^ .

Se conoce que el grado y extensión del daño auditivo está estrechamente relacionado con el nivel de presión sonora, su frecuencia, la duración y también con la susceptibilidad genética individual^(16)^. La hipoacusia neurosensorial inducida por ruido, por lo regular, está acompañada de síntomas tales como acúfenos, disminución de la capacidad de discriminación y distorsión de los sonidos^(17)^, manifestaciones susceptibles de ser evaluadas por métodos subjetivos.

A pesar de la relevancia de los temas expuestos anteriormente, la exposición al ruido de ocio no ha sido estudiada suficientemente en población adulta joven universitaria en ciudades de Latinoamérica. Colombia definió lineamientos de política para la promoción y gestión integral de la salud auditiva y comunicativa, desde 2014, con programas que enfatizan las acciones de promoción de la salud, prevención primaria, detección precoz y tratamiento ^(18)^ . Bajo estos lineamientos, este estudio investigó la participación de estudiantes de pregrado en actividades de ocio productoras de ruido con el fin de analizar la relación entre los niveles de exposición semanal al ruido recreacional y los síntomas de hipoacusia auto reportados, en jóvenes universitarios, en la ciudad de Barranquilla, Colombia.

## MÉTODO

El estudio tuvo aprobación del Comité de Ética en Investigación en el área de la Salud de la Universidad del Norte, según el acta No. 177 del 30 de agosto de 2018. Todos los participantes recibieron y aprobaron los términos del consentimiento informado.

Se realizó un estudio observacional transversal para estudiar los factores recreacionales de exposición a ruido y su asociación con el reporte de síntomas de hipoacusia en jóvenes universitarios de 18 a 29 años, matriculados en programas de pregrado de una universidad en la ciudad de Barranquilla, durante el primer semestre de 2019. Las covariables estudiadas fueron sexo y vivir en zona residencial ruidosa.

La muestra se calculó sobre una población total elegible de 13.431 estudiantes, con una proporción estimada de hipoacusia en jóvenes del 17% ^(2)^, una precisión de ± 3, un nivel de confianza del 95% y un efecto de diseño de 1.2. Al número estimado de 692 se adicionó un 5% por la eventual necesidad de descartar cuestionarios debido a la existencia previa de un problema auditivo de base, para un total de 730 estudiantes. Los sujetos se seleccionaron de forma aleatoria, estratificada por división académica, a partir de los cursos matriculados. Se envió mensaje de invitación con el enlace al cuestionario *online* a los estudiantes y a los coordinadores de los programas académicos con el fin de estimular la participación.

El cuestionario, elaborado en *Google Forms*, se estructuró en varias secciones. La primera, mostraba los términos del consentimiento informado seguido de un pregunta y casilla de aprobación, que en caso de no ser verificada impedía al estudiante continuar con el cuestionario. Había también una pregunta que indagaba el padecimiento de una alteración de la audición diagnosticada previamente (criterio de exclusión del estudio) y las relacionadas con los datos generales (edad, sexo, programa académico).

La segunda sección incluía el “Cuestionario de Exposición al Ruido” (CER), versión traducida y adaptada al idioma castellano, desde su versión original en inglés^(19)^, validada mediante juicio de expertos^(20)^. El CER comprendía a su vez dos partes: Parte A, que registraba la participación en actividades de ocio, el tiempo diario dedicado a cada una de ellas, el número de días por semana y la percepción subjetiva del ruido en una escala de 1 “muy silenciosa” a 5 “muy ruidosa”. Parte B, que indagaba por síntomas (tinnitus, dolor de oído y pérdida temporal de la audición ocasionadas por el ruido).

La tercera sección del cuestionario contenía los 11 ítems del cuestionario auditivo desarrollado por la empresa *Phonak®* (disponible en: https://www.yumpu.com/es/document/read/25302942/cuestionario-auditivo-phonak) para la autovaloración de la posible presencia y grado de severidad de pérdida auditiva.

### Medición de la exposición al ruido

De acuerdo con el método propuesto por Jokitulppo et al^(19)^, para la transformación de la valoración subjetiva de los encuestados sobre el nivel de ruido de las actividades evaluadas en el cuestionario, en estimaciones objetivas de la exposición al ruido recreativo se utilizó el nivel de ruido continuo equivalente máximo (LAeq dB) establecido en la literatura internacional (Tabla 1). Al valor en decibeles equivalentes de ruido continuo máximo en cada actividad, se le atribuyó la máxima puntuación de “5” (muy ruidosa) registrada en el cuestionario. Luego, con base en este valor se procedió a reducir 10 decibeles por cada punto por debajo de 5. Por ejemplo, en la categoría de dispositivos de audio personal, el valor de exposición máxima (5, muy ruidoso) equivale a 100 decibeles, es decir que para los valores inferiores contestados en el cuestionario la equivalencia de ruido en decibeles sería la siguiente: 4= 90 dB, 3=80 dB, 2=70 dB, 1=60 dB. Posteriormente, se calculó el promedio de exposición al ruido recreativo semanal y se comparó con el límite permisible para exposición ocupacional, establecido en 85 dBA, adoptado en Colombia^(21)^.

**Tabla 1:** Niveles sonoros equivalentes para las actividades de ocio estudiadas

**Actividad**	**Intervalo de intensidad**	**Valor máximo de LAeq**	**Referencia**
Tocar en un grupo o banda	90-135	105	MCR, 1986; Salamivalli, 1990; Drake-Lee, 1992
Escuchar música clásica	75-114	105	Axelsson et al, 1981a; Jansson y Karsson, 1983; Clark, 1991; Royster y Royster, 1991;
Máquinas electrónicas de arcade	73-111	100	Plakke, 1983; Clark, 1991
Equipo de música con altavoces	70-100	100	Axelsson et al, 1981a; Salmivalli, 1990
Equipo de música con auriculares	85-120	100	Axelsson et al, 1981a; Salmivalli, 1990
Reproductor de música personal	53-115	100	Kurss y Findlay, 1974; Catalano y Levin, 1985; Lees et al, 1985; Wong et al, 1990; Clark, 1991; Airo et al, 1995
Deportes de motor	70-112	105	Axelsson et al, 1981a; Clark, 1991
Discotecas y conciertos	84-125	105	Fearn, 1972; Ulrich et al, 1974; Axelsson et al, 1981a; MRC, 1986; Salmivalli, 1990; Göthe, 1992
Herramientas caseras	60-115	105	Axelsson et al, 1981; Salmivalli, 1990; Clark, 1991
Tiro		105	Estimado

Fuente: Jokitulppo et al. ^(19)^

**Subtítulo:** LAeq = nivel de ruido continuo equivalente

### Medición del grado de pérdida auditiva

Para clasificar el posible grado de pérdida auditiva, con base en las 11 preguntas del cuestionario auditivo, por cada respuesta afirmativa se le asignó 1 punto (rango de 0 a 11 puntos). Luego del conteo del total de preguntas afirmativas por participante, se conformaron dos grupos: Grupo “con probable pérdida auditiva” de algún grado (4 o más puntos) y grupo “sin sospecha de pérdida auditiva” (0-3 puntos).

La asociación entre los síntomas de hipoacusia del cuestionario CER y el posible grado de pérdida auditiva con el nivel de exposición al ruido recreativo equivalente (“mayor de 85 dB” versus “menor o igual a 85 dB”) se evaluó mediante el *odds ratio*, el intervalo de confianza al 95% y la significancia estadística establecida en p < 0.05, mediante la prueba de Chi cuadrado (*X^2^* ). El análisis se realizó en Epi-info versión 7.2

## RESULTADOS

Todos estudiantes invitados a participar respondieron el cuestionario. De las 730 encuestas, se excluyeron 18 debido al reporte de problemas auditivos previos, para un total de 712 sujetos incluidos en el análisis. Poco más de la mitad de los encuestados era de sexo femenino (59,6%) y en ambos sexos el grupo de edad predominante fue el de 18 a 21 años (87%), sin diferencias significativas en la distribución por edad y sexo (p>0.05). El 25,2% eran de los programas de Ingenierías, 18,3% de Ciencias de la Salud, 18.1% de Humanidades, 14.8% de la Escuela de Negocios, 12,29% de Derecho y Ciencias Políticas, 5,3% de Arquitectura, 3,7% de Ciencias Básicas, 1,2% de Educación y solo 0,7% de Música. El 50,28% (358/712) vivía en zona residencial ruidosa.

### Exposición al ruido recreativo

Las actividades recreativas evaluadas a las que los encuestados dedicaban más tiempo por semana fueron: uso de dispositivos portátiles (18 h), escuchar TV (10,3 h) y asistencia a discotecas (2,2 h) a la semana (Figura 1). Las actividades de ocio percibidas como más ruidosas por los universitarios fueron las discotecas, conciertos, bares, tiro y uso de dispositivos de audio portátiles personales (Figura 2).

**Figura 1:** Tiempo dedicado a las actividades recreativas analizadas (promedio de horas por semana)

**Figura 2:** Estimación subjetiva de la intensidad sonora de las actividades recreativas estudiadas, expresadas en escala de 1 “muy silenciosa” a 5 “muy ruidosa”

La exposición semanal al ruido calculada a partir del total de la muestra, en relación con todas las actividades, el tiempo dedicado a cada una de ellas y la intensidad del ruido estimado para cada actividad muestra que la mediana de exposición fue de 87 dBA. Al analizar la distribución de la exposición por sexo (Figura 3) se encontró que un 58% de las mujeres, en contraste con un 47% de los hombres están expuestos a niveles de ruido por encima del límite de riesgo de 85 dBA establecidos en la norma colombiana^(20)^ lo que revela diferencias significativas (p<0,05).

**Figura 3:** Distribución de la exposición semanal al ruido recreativo por sexo. Los resultados fueron calculados para todos los sujetos en relación a su exposición a las actividades estudiadas, el tiempo dedicado a cada una y el nivel de intensidad

### Síntomas de hipoacusia

De los síntomas indagados en el cuestionario CER, el zumbido en los oídos fue el más referido (72%), seguido por el dolor de oídos (44%), y la pérdida temporal de la audición (23%). La Tabla 2 muestra que la exposición a niveles de ruido por encima del umbral de riesgo para daño auditivo se asoció de forma significativa a la presencia de tinnitus y al dolor de oídos (p<0,05).

**Tabla 2:** Asociación de los síntomas auditivos reportados con el nivel de exposición al ruido recreativo

	**Zumbido**			**Dolor**			**Pérdida de la audición por ruido**		
	**n (%)**	**OR (IC_95%_)**	**Valor p** *	**n (%)**	**OR (IC_95%_)**	Valor p*	**n (%)**	**OR (IC_95%_)**	Valor p*
***>85 dB***	309 (60)	1,78 (1,28-2,4)	<0,05	200(63)	1,73 (1,27-2,3)	<0,05	93(56)	0,99 (0,7-1,4)	>0,05
**≤85 dB**	206 (40)			115(37)			73(44)		
**Total**	515 (100)			315(100)			166(100)		

* Prueba estadística: X^2^

**Subtítulo:** n= número de casos; OR= *odds ratio*; IC= intervalo de confianza

En la Tabla 3 se observa que de los 11 síntomas de hipoacusia evaluados mediante el Test Auditivo, los cinco más frecuentemente reportados fueron “problemas para entender conversaciones cuando hay ruido en ambiente o personas hablando“ (55%), “necesidad de pedir a otros que le repitan” (49%), “no entender lo que se le dice cuando va en coche o está en ambiente ruidoso” (46%), “sentir estrés o cansancio al escuchar algo por largo tiempo” (40%) y “colocar el volumen de la TV más alto que otros para oír bien” (39%).

**Tabla 3:** Frecuencia de síntomas auditivos en la población total (Test Auditivo) y número de síntomas por participante

.	n (%)	IC 95%
**Síntomas de hipoacusia:** Problemas para entender conversaciones cuando hay ruido en ambiente o personas hablando.	394 (55)	51,6-59,0
Necesita pedir a otros que le repitan lo que han dicho	353 (49)	45,8-53,3
Cuando va en un coche o ambiente ruidoso no entiende lo que se dice	327 (46)	42,2-49,6
Siente estrés o cansancio al escuchar algo por largo tiempo	285 (40)	36,3-43,7
Coloca volumen TV más alto que otros para oír bien.	276 (39)	35,142,4
Piensa que otros están murmurando o hablando poco claro	245 (34)	30,8-37,9
Necesita sentarse cerca a otros para escuchar lo que le dicen.	209 (29)	25,9-32,7
Problemas para oír a otros cuando no están de frente.	166 (23)	20,1-26,5
Evita fiestas o eventos por mucho o no poder oír bien.	148 (21)	17,7-23,8
Recibe preguntas acerca de posibles problemas auditivos	133 (19)	15,7-21,6
Dificultad para identificar procedencia de sonidos.	105 (15)	12,0-17,4
**Frecuencia de síntomas de hipoacusia en una misma persona:**		
1 a 3 síntomas	306 (43)	39,3-46,7
4 a 6 síntomas	263 (37)	33,3-40,5
7 a 11 síntomas	93 (13)	10,5-15,6
0 síntomas	50 (7)	5,5-8,9

**Subtítulo:** n= número de casos; IC= intervalo de confianza

La clasificación del grado estimado de probable pérdida de la audición, según el número de síntomas (Tabla 3) muestra que poco menos de la mitad se ubica en el grupo de 1-3 síntomas, considerado como “sin sospecha de pérdida auditiva” (43%) mientras que el resto clasifica para el grupo “con probable pérdida auditiva de algún grado”.

### Variables demográficas y probable pérdida auditiva

Aunque las mujeres presentaron 1,3 veces más chance de tener probable pérdida auditiva que los hombres, no se encontró una asociación significativa (p>0,05). Asimismo, el “vivir en zona residencial ruidosa” tampoco se asoció con la posibilidad de tener probable pérdida auditiva (p>0,05), (Tabla 4)

**Tabla 4:** Asociación entre el sexo, lugar de residencia y exposición al ruido recreativo con la presencia de probable pérdida auditiva de algún grado

	Con probable pérdida auditiva	Sin Pérdida auditiva	**OR (IC_95%_)**	**Valor p** *
	n= 352	n=360		
Sexo				
				
Femenino	222	203		
			1,32 (0,97-1,78)	0,069
Masculino	130	157		
				
Zona residencial ruidosa				
				
Si	176	182	0,9 (0,72-1,31)	0,882
				
No	176	178		
				
Intensidad Ruido recreativo				
				
> 85 dBA	217	182	1,57 (1,16-2,11)	0,002
				
≤ 85 dBA	135	178		
				

* Prueba estadística: X^2^

**Subtítulo:** OR= *odds ratio*; IC= intervalo de confianza

### Exposición al ruido recreativo y grado estimado de probable pérdida auditiva

Los estudiantes que estaban expuestos a niveles semanales de ruido recreativo por encima del límite permisible presentaron 1,57 (IC_95%_ = 1,16-2,11) veces más chance de tener 4 o más síntomas de hipoacusia; es decir, probable pérdida auditiva de algún grado (Tabla 4). Esta asociación se mantuvo en el análisis ajustado por sexo (ORa 1,53; IC_95%_= 1,12-2,09).

## DISCUSIÓN

Este es el primer estudio que indaga la prevalencia de síntomas de hipoacusia auto reportada, en relación con la exposición al ruido recreativo y ambiental en estudiantes de pregrado de 18-29, en una ciudad capital de la región Caribe colombiana.

Un hallazgo relevante del estudio es el tiempo de dedicación semanal a actividades recreativas productoras de ruido, en particular, el uso de dispositivos personales de audio y la televisión, similar a lo reportado para jóvenes de Europa^(12,18-19)^, Asia^(9)^ , Estados Unidos^(7)^ y Latinoamérica^(8,22)^ .

Se ha estudiado que los dispositivos personales de audio generan niveles de presión de sonido tan altos como 124 dBA en reproductores de CDs ^(12-13,23)^; sin embargo, solo la mitad de los jóvenes universitarios estudiados perciben esta actividad como muy ruidosa o poco ruidosa, a diferencia de lo reportado en jóvenes asiáticos^(9)^

Igualmente, la asistencia a discotecas 2.3 hs semanales, declarada por los participantes, constituye un factor de exposición al ruido muy importante en el medio; no solo por generar mayores niveles de intensidad ^(21)^, sino por la posibilidad de haber sido subestimada en el estudio, debido a que el cuestionario CER indaga por la asistencia semanal y no para tiempos más amplios.

A diferencia de los jóvenes europeos ^(18-19)^, en la población universitaria encuestada fue infrecuente la exposición relacionada con el tocar en un grupo de música; probablemente debido a que los estudiantes de música estuvieron subrepresentados en este estudio por ser éste un programa de reciente apertura en la universidad.

La mediana de exposición total semanal al ruido en este estudio (87 dBA) estuvo 14 dBA por encima de la registrada para población adulta nórdica^(24)^ y 2 dBA arriba del estimado en los adolescentes españoles^(19)^. Poco más de la mitad de la población estudiada está expuesta a un nivel de ruido recreativo, por encima del límite de riesgo establecido internacionalmente lo que implica un riesgo de sufrir pérdida auditiva en el corto, mediano o largo plazo. Esta frecuencia es inferior a la encontrada en Alemania (25%) debido a que en este país además de la medición subjetiva se utilizó la audiometría como método objetivo^(12)^; mientras que sí coinciden con los reportado en estudios anteriores para adolescentes hasta los 19 años ^(18)^ y adultos jóvenes finlandeses^(24)^ y para adolescentes españoles^(19)^, que utilizaron la misma metodología. Asimismo, es consistente con las estimaciones globales de la OMS para adolescentes y jóvenes de 12-35 años^(25)^. El alto porcentaje de exposición a niveles peligrosos de ruido recreativo en distintos países sugiere deficiencias en los programas de promoción de la salud auditiva, a pesar de la normatividad existente.

La alta proporción de jóvenes universitarios con probable hipoacusia de algún grado encontrada en este estudio podría ser explicada, en parte, por la acumulación de exposiciones al ruido recreativo, durante la etapa previa de adolescencia, toda vez, las actividades más reportadas (escuchar música en dispositivos de audio a alto volumen y ver televisión) son hábitos que suelen permanecer durante la vida o inclusive llegar a incrementarse. Según el estudio audiológico de seguimiento a 4 años en adolescentes, realizado en la ciudad de Córdoba, Argentina, el nivel umbral auditivo promedio tiende a incrementarse en el tiempo, en paralelo al incremento de su participación en actividades recreativas musicales, asistencia a discotecas y a conciertos^(6)^.

El tinnitus, identificado por la percepción de zumbido en los oídos, fue la molestia auditiva más reportada en el presente estudio y merece particular atención, al ser el deterioro auditivo su principal factor de riesgo^(26)^. Los hallazgos de una revisión sistemática^(27)^ indican que existe controversia en los efectos del tinnitus en la memoria de trabajo y en la atención y además señalan una alta probabilidad de sesgo en los estudios publicados; no obstante, estas funciones son de capital importancia en el desempeño académico de los universitarios, por lo que deberán ser estudiados en futuras investigaciones.

El análisis de los factores asociados con la presencia de síntomas de hipoacusia aislados y el tener varios síntomas simultáneamente sugestivos de probable pérdida auditiva de algún grado, indica que no hubo diferencias significativas en presencia de estos síntomas de hipoacusia entre los participantes que declararon estar expuestos y no expuestos a zonas residenciales cercanas a sitios de construcción o mucho tráfico; posiblemente este hallazgo se explica por la contaminación auditiva descritos en la ciudad de Barranquilla, ocasionadas por diversas fuentes de ruido^(28)^.

Este estudio aporta nueva evidencia del riesgo que supone la exposición a niveles de ruido recreativo por encima del límite de 85 dBA para la población joven universitaria, al observarse un mayor chance de presentar, simultáneamente, varios síntomas sugestivos de probable pérdida auditiva de algún grado. Estos hallazgos urgen la necesidad implementación de acciones de promoción y prevención desde la academia. En el ámbito universitario, estas acciones se enmarcan en recomendaciones de políticas internacionales y nacionales para la promoción y cuidado de la salud. Las primeras, fundamentadas en el concepto de universidad saludable o universidad promotora de salud, definida como “*aquélla que incorpora la promoción de la salud, a su proyecto educativo y laboral, con el fin de propiciar el desarrollo humano y mejorar la calidad de vida de quienes allí estudian o trabajan y, a la vez, formarlos para que actúen como modelos o promotores de conductas saludables a nivel de sus familias, en sus futuros entornos laborales y en la sociedad en general*” ^(29)^ y las segundas, a partir de los lineamientos nacionales para la promoción y gestión integral de la salud auditiva y comunicativa^(18)^.

Una limitante de este estudio es el desconocimiento de la verdadera capacidad auditiva de los participantes, debido a la medición subjetiva realizada. Según señalan revisiones acerca de la confiabilidad de las encuestas de pérdida auditiva auto reportada, la medición subjetiva tiene sus imprecisiones: subestima la presencia de pérdida auditiva en los adultos mayores, y tiende a sobrestimarla en los más jóvenes^(4)^; por lo tanto, es insuficiente para identificar con exactitud a los individuos con déficit auditivo. Sin embargo, las preguntas expuestas en el cuestionario auditivo permiten, desde el escenario académico, conocer los diferentes síntomas que podrían relacionarse con tales alteraciones. En particular, las preguntas acerca de la dificultad para entender una conversación en ambientes ruidosos son útiles para detectar una posible pérdida auditiva oculta o sinaptopatía coclear, que puede pasar desapercibida en un audiograma^(30)^. Otra limitante del estudio fue la no evaluación de la frecuencia de los síntomas de hipoacusia.

Según la revisión de la literatura realizada por los autores, existe un vacío de publicaciones de estudios sobre hipoacusia en población universitaria en Colombia, situación que limitó la posibilidad de comparación de los resultados obtenidos en Barranquilla con la situación en otras ciudades capitales del país. En este sentido, este estudio es útil como precedente para futuras investigaciones en donde se establezca la agudeza auditiva por métodos de evaluación subjetivos y objetivos y se correlacione con las características ambientales, recreacionales y del comportamiento. Adicionalmente, sirve de base para la elaboración de programas de sensibilización y educación en salud auditiva que fomenten la adopción de conductas protectoras ante posibles problemas auditivos en escenarios universitarios y se recomienda el tamizaje de la audición en el examen médico a los estudiantes que entran por mi primera vez a la universidad, así como conductas correctivas ante problemas de audición instaurados.

## CONCLUSIONES

La estimación subjetiva de la intensidad de exposición al ruido y los síntomas de hipoacusia en jóvenes universitarios sirve de tamizaje para la identificación temprana de problemas auditivos y provee información de base poblacional para la toma de decisiones. La gran prevalencia de síntomas auditivos reportados en este estudio refleja la relevancia del problema. Si bien los universitarios encuestados identifican las actividades recreativas y ambientales más ruidosas declaran entre sus actividades diarias preferidas el uso de dispositivos portátiles de audio y la asistencia a discotecas, que los exponen a niveles de intensidad promedio superiores a los 85 dB, lo que conlleva un riesgo de padecer trastornos auditivos severos.

Este estudio provee nueva evidencia de la asociación entre la prevalencia de síntomas auditivos y la exposición continua a niveles de ruido promedios mayores a 85 dB en jóvenes universitarios. Es preciso incorporar o intensificar la promoción de la salud auditiva, en ambientes universitarios, que sensibilicen sobre las conductas que predisponen al desarrollo de la hipoacusia o empeoran su condición de base. Simultáneamente, se recomienda realizar programas de detección temprana más objetivos, a través de los servicios de bienestar universitario, en articulación con la red de servicios de salud. De esta forma, se podrá facilitar la continuidad en la atención de los jóvenes en riesgo de hipoacusia, que garantice el tratamiento oportuno, limitando el daño.
